# Poor Trail Making Test Performance Is Directly Associated with Altered Dual Task Prioritization in the Elderly – Baseline Results from the TREND Study

**DOI:** 10.1371/journal.pone.0027831

**Published:** 2011-11-16

**Authors:** Markus A. Hobert, Raphael Niebler, Sinja I. Meyer, Kathrin Brockmann, Clemens Becker, Heiko Huber, Alexandra Gaenslen, Jana Godau, Gerhard W. Eschweiler, Daniela Berg, Walter Maetzler

**Affiliations:** 1 Hertie Institute for Clinical Brain Research, Department of Neurodegeneration, Center of Neurology, University of Tuebingen, Tuebingen, Germany; 2 DZNE, German Center for Neurodegenerative Diseases, University of Tuebingen, Tuebingen, Germany; 3 Department of Psychiatry and Psychotherapy, University Hospital Tuebingen, Tuebingen, Germany; 4 Robert-Bosch-Hospital, Department of Clinical Gerontology, Stuttgart, Germany; 5 Geriatric Center at the University Hospital Tuebingen, Tuebingen, Germany; Federal University of Rio de Janeiro, Brazil

## Abstract

**Background:**

Deterioration of executive functions in the elderly has been associated with impairments in walking performance. This may be caused by limited cognitive flexibility and working memory, but could also be caused by altered prioritization of simultaneously performed tasks. To disentangle these options we investigated the associations between Trail Making Test performance—which specifically measures cognitive flexibility and working memory—and dual task costs, a measure of prioritization.

**Methodology and Principal Findings:**

Out of the TREND study (Tuebinger evaluation of Risk factors for Early detection of Neurodegenerative Disorders), 686 neurodegeneratively healthy, non-demented elderly aged 50 to 80 years were classified according to their Trail Making Test performance (delta TMT; TMT-B minus TMT-A). The subjects performed 20 m walks with habitual and maximum speed. Dual tasking performance was tested with walking at maximum speed, in combination with checking boxes on a clipboard, and subtracting serial 7 s at maximum speeds. As expected, the poor TMT group performed worse when subtracting serial 7 s under single and dual task conditions, and they walked more slowly when simultaneously subtracting serial 7 s, compared to the good TMT performers. In the walking when subtracting serial 7 s condition but not in the other 3 conditions, dual task costs were higher in the poor TMT performers (median 20%; range −6 to 58%) compared to the good performers (17%; −16 to 43%; p<0.001). To the contrary, the proportion of the poor TMT performance group that made calculation errors under the dual tasking situation was lower than under the single task situation, but higher in the good TMT performance group (poor performers, −1.6%; good performers, +3%; p = 0.035).

**Conclusion:**

Under most challenging conditions, the elderly with poor TMT performance prioritize the cognitive task at the expense of walking velocity. This indicates that poor cognitive flexibility and working memory are directly associated with altered prioritization.

## Introduction

The application of dual task paradigms to evaluate the role of executive functioning during walking is generally well-accepted. Commonly used dual task paradigms include a walking task combined with a simultaneously performed non-walking task, and it has been suggested that some but not all combinations of walking with a non-walking task contribute to disturbed gait and, consecutively, to increased risk for falls with increasing age [1], [2], [3], [4]. Walking is associated with higher-level cognitive resources, in particular with executive functions such as cognitive flexibility and working memory, which both deteriorate with increasing age [4]. Not surprisingly, an association between cognition and walking speed among elderly people has been demonstrated [5], and a decline in executive functions is one of the determinants of walking impairment that is often observed in older persons [6], [7].

Another important part of executive functioning, and aspect in dual tasking is prioritization of one task over the other, following the motivation to minimize danger and maximize pleasure [4]. Healthy adults prioritize stability of gait when walking and simultaneously performing a cognitive task [2], [8], [9]. This seems to be different in Parkinson disease patients [10], in elderly fallers [11], and in Parkinsonian patients who fall regularly [12]. They have an increased probability to use a “posture second” strategy, and to prioritize the cognitive task at the expense of the stability of walking. However, most of these studies put their focus on the evaluation of the walking task but not on the non-walking task, thus knowledge about dual-task behaviour of older subjects, in particular with regard to non-walking tasks, is still limited. In addition, most of the studies used paradigms performed with habitual speed but not with maximum speed. This may lead to an oversight of subtle differences and false negative results [7], [13], [14].

To the best of our knowledge, the association of cognitive flexibility and working memory with dual tasking prioritization has never been investigated in a large cohort of healthy elderly. In this study, this was tested in 686 non-demented healthy older persons by evaluating the performance of walking and non-walking tasks under challenging conditions. The Trail Making Test (TMT) performance as a measure of cognitive flexibility and working memory was used to divide the cohort into a good, an intermediate, and a poor performance group.

## Methods

### Ethics

The study protocol was approved by the ethical committee of the Medical Faculty of the University of Tuebingen (Nr. 90/2009BO2), and all subjects provided written informed consent.

### Objective

The primary objective of the study was to test whether poor performance on the Trail Making test as a measure of cognitive flexibility and working memory is associated with altered prioritization under dual tasking behaviour in a large cohort of older healthy persons. Secondary aim was to exploratively analyze direction and degree of prioritization in the defined subgroups.

### Subjects

In the baseline assessment of the TREND study (**T**übinger evaluation of **R**isk factors for **E**arly detection of **N**eurodegenerative **D**isorders) 715 subjects aged 50–80 years with or without risk factors for Parkinson's and Alzheimer's disease (hyposmia, depression, REM sleep behavior disorder) were investigated prospectively in 2009 and 2010. A detailed description of the study outline, including inclusion and exclusion criteria, and baseline assessments, is given in (Berg et al., submitted). In brief, all subjects were pre-screened via telephone interview, and were excluded if they reported a history of psychiatric diseases (other than primary depression), dementia, epilepsy, stroke, multiple sclerosis, encephalitis and malignancies, intake of antipsychotics and other drugs that are able to promote Parkinsonian symptoms, and inability to walk without aids or assistance. In addition, disorders that could allow only incomplete study performance, such as paresis, sensory loss or significant impairment of vision or hearing all lead to primary exclusion of the subjects from the study.

From the investigated 715 subjects, a total of 29 subjects were excluded from this analysis due to the following reasons: Eleven met the criteria for Parkinson disease according to the UK Brain Bank Society criteria, eight had incomplete TMT data, five had negative delta TMT values, and five had a Mini-Mental Score Examination score <25. For demographic characteristics see table 1.

**Table 1:** Demographics and clinical assessments, and Trail Making Test performance.

*Performers*	good (N = 227)	intermediate (N = 226)	poor (N = 233)	P	Total cohort (N = 686)
Age [years]	61 (50–78)	64 (50–80)*	66(50–80)* #	<0.001	64 (50–80)
Male [%]	45.8	49.1	46.4	0.75	47.1
Education period [years]	15 (7–20)	14 (8–20)	13 (8–20)* #	<0.001	14 (7–20)
MMSE (0–30)	29 (26–30)	29 (25–30)*	29 (25–30)* #	<0.001	29 (25–30)
BDI (0–63)	6 (0–29)	6 (0–38)	7 (0–42)	0.22	6 (0–42)
Weight [kg]	71 (48–125)	74 (49–150)	75 (45–117)	0.31	73 (45–150)
Height [m]	1.70 (1.54–1.92)	1.71 (1.54–2.00)	1.70 (1.48–1.90)	0.38	1.70 (1.48–2.00)
TMT-A [s]	33 (16–90)	35 (20–88)	36 (15–100)*	0.006	35 (15–100)
TMT-B [s]	60 (34–97)	80 (58–140)*	120 (82–300)* #	<0.001	83 (34–300)
Delta TMT [s]	26 (0–35)	47 (36–58)*	80 (59–261)* #	<0.001	47 (0–261)

Good performers were defined as having a delta TMT score of less than 36 seconds, intermediate performers as having a delta TMT score of 36–58 s, and poor performers as having a delta TMT score higher than 58 s. Data are presented with median and range. P-values were assessed using the Kruskal Wallis test. P-values<0.05 were considered significant.

*p<0.05 compared to good performers;

# p<0.05 compared to intermediate performers. BDI, Beck's Depression Inventory; BMI, Body Mass Index; MMSE, Mini-Mental State Examination; TMT, Trail Making Test.

### Single and dual task procedures

All subjects performed four single task trials: walking with habitual speed, walking with maximum speed, checking boxes with maximum speed, and subtracting serial 7 s with maximum speed. During the box-checking task, participants held a clipboard in their non-dominant, and a pen in the other hand. Then they had to mark each of 32 boxes with a cross on a sheet of paper with a pencil. The instruction was as follows: “Please mark each of the boxes on the sheet of paper with a cross as fast as you can.” There was no instruction about where to start and to end with, and about the order of crossing. During the subtracting task, subjects had to subtract serial 7 s from a randomly chosen three-digit number until 10 subtractions were completed. The instruction was as follows: “Please subtract serial 7 s as fast as you can from the number I will shortly tell you, until I will interrupt you.”

In the two dual task assessments, subjects performed both walking with maximum speed and checking boxes with maximum speed, and walking with maximum speed and subtracting serial 7 s with maximum speed. Instructions were as follows: “Please walk as fast as you can, do not run, do not risk falling, and mark each of the boxes on the sheet of paper with a cross as fast as you can,” and “Please walk as fast as you can, do not run, do not risk falling, and subtract serial 7 s as fast as you can from the number I will shortly tell you.” A randomly chosen three-digit number different from the number used for the single task assessment was told to the participant directly before the start sign was given. No hint for prioritization on any task was given, to omit an external influence on the prioritization process [15]. All assessments were performed in an at least 1.5 meters wide corridor allowing obstacle-free 20 meter walks.

Time was taken with a stopwatch and documented by the examiner, as were number of checked boxes, number of subtractions, and number of subtraction errors.

### Cognitive assessment

The Trail Making Test (TMT) is a widely used paper-and-pencil task that evaluates the executive functions cognitive flexibility and working memory [7], [16]. The TMT consists of two parts: On TMT Part A subjects have to connect numbers from 1 to 25, which are randomly spread over a sheet of paper, in ascending numerical order. On part B, participants are asked to connect randomly spread numbers (from 1 to 13) and letters (from A to L) in alternating numeric and alphabetical order (1-A-2-B-3-C-…-13-L). In case of an error the examiner draws the attention of the participant to the error, so that the participant completes the task without errors (at the expense of additional time) [17]. TMT performance was calculated taking the time needed to perform TMT-B minus time needed for TMT-A. This delta TMT value “removes” eventual bias due to differences in upper extremity motor speed, simple sequencing, visual scanning, and psychomotor functioning [7], [16], [17], [18].

### Data processing and statistical analysis

Data were analysed with JMP software (version 8.0.2, SAS), and are presented with median and range if not otherwise indicated. Subjects with delta TMT values>58 s were defined as poor performers (lowest tertile, N = 233), those with 36–58 s as intermediate performers (N = 226), and those with <36 s as good performers (highest tertile, N = 227). Demographic and basic clinical variables of the groups were compared by use of the Kruskal Wallis test (or, in case of categorical data the Chi square test), and post-hoc Wilcoxon test (Chi square test) (table 1). Outcome variables (table 2 and table 3) were corrected for age (R^2^≤13%, with high values for the box checking task, and negligible values for subtracting serial 7 s), gender, education level (R^2^≤5%), Mini-Mental State Examination score (R^2^≤4%) and Becks Depression Inventory score (R^2^≤4%) by use of a logistic regression model, and significance of each model effect was assessed by the likelihood ratio. Differences were considered significant at p<0.05 (two-sided). The parameters “box-checking speed” and “subtracting performance” were defined as numbers of checked boxes / subtractions over time needed for the task (seconds). Dual task costs were calculated using the following formula according to [12], [19]:
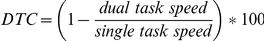
This formula gives information about the percentage of change compared to the single task value. A positive value indicates a decrease of speed. The parameter “subtraction errors” was defined by the proportion of people among a cohort which made at least one error.

**Table 2:** Single and dual task results.

*Performers*	good	intermediate	poor	P
***Single task conditions***				
Walking with habitual speed [m/s]	1.39 (0.87–1.99)	1.38 (0.85–2.02)	1.36 (0.93–1.81)	0.85
Walking with maximum speed [m/s]	1.75 (1.04–2.51)	1.70 (1.06–2.53)	1.64 (1.06–2.59)	0.20
Checking boxes [1/s]	1.64 (1.00–2.30)	1.56 (0.99–2.37)	1.48 (0.81–2.81)	0.03
Subtracting [1/s]	0.41 (0.13–1.08)	0.36 (0.09–0.90)*	0.32 (0.07–0.93)*	<0.001
At least one subtraction error (proportion of cohort, %)	24.5	37.0*	43.8*	<0.003
***Dual task conditions***				
Walking when checking boxes [m/s]	1.53 (0.88–2.20)	1.47 (0.85–2.20)	1.42 (0.83–2.30)	0.08
Checking boxes when walking [1/s]	1.46 (0.60–2.59)	1.39 (0.55–2.44)	1.32 (0.46–3.66)	0.42
Walking when subtracting [m/s]	1.44 (0.92–2.15)	1.40 (0.68–2.53)	1.30 (0.74–2.06)* #	<0.001
Subtracting when walking [1/s]	0.48 (0.07–1.12)	0.45 (0.07–1.03)*	0.37 (0.05–1.05)* #	0.004
At least one subtraction error (proportion of cohort, %)	27.5	40.5*	42.2*	0.003

Data are presented with median and range. P-values were calculated using a logistical regression model and the likelihood ratio, with correction for age, gender, education level, Mini Mental Status Examination score and Becks Depression Inventory score.

*p<0.05 compared to good performers;

# p<0.05 compared to intermediate performers.

**Table 3:** Dual task costs.

*Performers*	good	intermediate	poor	P
***Dual task costs***				
Walking when checking boxes [%]	11.0 (−5.4–35.8)	10.9 (−76.0–65.0)	12.8 (−40.3–58.1)	0.11
Checking boxes when walking [%]	10.4 (−83.2–53.0)	10.2 (−66.8–54.1)	12.0 (−121.0–68.5)	0.51
Walking when subtracting [%]	16.7 (−16.1–43.4)	17.3 (−38.0–58.2)	19.8 (−6.3–58.6)* #	<0.001
Subtracting when walking [%]	−15.9 (−156.9–75.4)	−22.6 (−227.4–60.5)	−17.3 (−190.1–76.8)	0.20
At least one subtraction error (proportion of cohort, %)	3.0	3.5	−1.6*	0.07

Data are presented with median and range. P-values were calculated using a logistical regression model and the likelihood ratio, with correction for age, gender, education level, Mini Mental Status Examination score and Becks Depression Inventory score. Difference of subtraction errors were calculated with Chi square test.

*p<0.05 compared to good performers;

# p<0.05 compared to intermediate performers.

## Results

Six hundred eighty-six persons were included in the analysis. Details about demographic and clinical variables are supplied in table 1. Among the investigated single tasking conditions, habitual walking speed, maximum walking speed, and checking boxes speed were not significantly associated with TMT performance. Only subtracting serial 7 s speed (good versus poor performers, p<0.001) was associated with TMT performance. In addition, more poor than good performers made at least one error when subtracting serial 7 s (p<0.001, table 2).

Under dual tasking conditions, checking boxes speed when walking with maximum speed and maximum walking speed when checking boxes were not significantly associated with TMT performance. Subtracting serial 7 s speed when walking with maximum speed (good versus poor performers, p<0.001), and maximum walking speed when subtracting serial 7 s (good versus poor performers, p<0.001) were associated with TMT performance. More poor performers than good performers made at least one error when subtracting serial 7 s (p = 0.002). Details are supplied in table 2.

**Figure 1:**
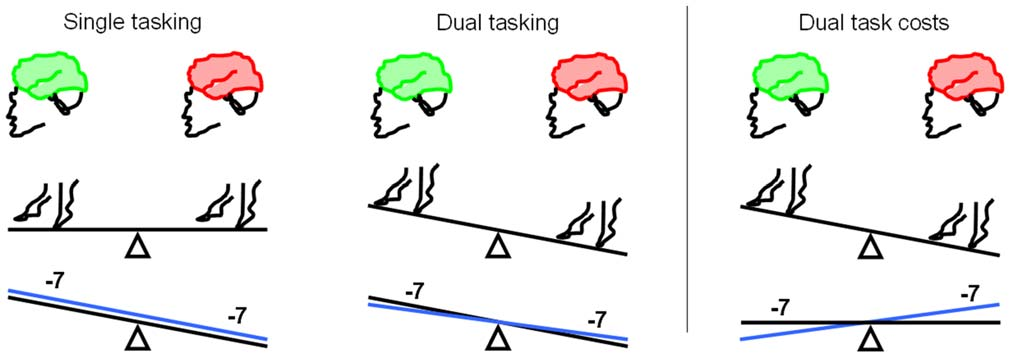
Good versus poor trail making test performers: overview of differences in single and dual tasking behaviour, and in dual task costs. Under challenging conditions, older persons with good Trail Making Test (TMT) performance (good cognitive flexibility and working memory; green brain, left) perform comparably well as poor TMT performers (red brain, right) regarding walking velocity, but better on a cognitive single task which is associated with executive functioning (subtracting serial 7 s). The black line represents subtraction velocity and the blue line subtraction errors. Under dual tasking conditions (maximum walking speed and subtracting serial 7 s with maximum speed), poor performers walk more slowly than good TMT performers. Velocity of the serial −7 s task is still lower in the poor TMT performers, but the difference in number of errors between good and poor TMT performers is smaller than under single task conditions. This is also reflected in the dual task costs: Regarding the walking task, dual task costs are higher in poor TMT performers than in good TMT performers. However, dual task costs of velocity of the serial −7 s task are not significantly different, and dual task costs of subtraction errors are even lower in poor TMT performers than in good TMT performers. This demonstrates that older persons with poor cognitive flexibility and working memory prioritize differently to those with good cognitive flexibility and working memory when performing a challenging dual task paradigm with a walking and a cognitive task.

Dual task costs were not significantly different between the investigated groups for checking boxes speed and for subtracting serial 7 s speed, respectively. Also dual task costs at maximum walking speed when checking boxes was not significantly different between the groups. Dual task costs at maximum walking speed when subtracting serial 7 s was higher in the poor TMT performance group (good versus poor performers, p<0.001). In addition, among the good and intermediate performers, groups proportions that made an error when subtracting serial 7 s were higher under the dual task condition than under the single task condition. Among the poor performers, the proportion that made a calculation error when subtracting serial 7 s was *lower* under the dual task condition than under the single task condition (good vs. poor performers, p = 0.035). Detailed data are shown in table 3. A schematic overview of the abovementioned results is given in figure 1.

## Discussion

The main new finding of this representative study of non-demented healthy elderly is that under most challenging dual tasking conditions, subjects with poor cognitive flexibility and working memory show higher dual task costs of the walking task but perform better in the subtracting serial 7 s task, compared to older persons with good cognitive flexibility and working memory. Thus, older persons with poor cognitive flexibility and working memory do not show a *comparably* increased slowing of motor and cognitive processes in dual tasking as one may expect, but prioritize the cognitive task at the expense of the gait task. As healthy adults prioritize stability of gait when walking and simultaneously performing a cognitive task [2], [8], [9], our findings argue for an altered prioritization process in older persons with poor cognitive flexibility and working memory. This is, to the best of our knowledge, the first study demonstrating a direct link between these executive functions in a considerably large cohort of healthy elderly.

This study used a similar approach as a former study [7]. In this former study the authors found that poor TMT performance was associated with poor performance when walking on an obstacle course. Despite some relevant differences regarding the study population between the former and our study (e.g., age at study inclusion was 64 years in our study, and 75 years in the former one; education period 14 versus 6 years; Mini-Mental State Examination score 29 versus <26 points; delta TMT of the poor performers in this study >58 s, of the *good* performers in the former study <78 s) and differing study outlines (no dual tasking paradigms in the former study) some aspects are comparable: All three TMT performance groups of the former study used similar speed when walking with habitual speed, and differences between the groups were only observable under the more complex walking situation. With regard to the abovementioned association between prioritization and dual task behaviour, it is tempting to speculate that those subjects who performed poor on the obstacle course in the former study would also differ from the good TMT performers regarding their prioritization pattern.

Dual task costs are defined as adaptation processes during the simultaneous performance of two tasks in comparison to perform each task solely. It is a measure of the effect of divided attention. As dividing attention is considered an executive function, we conclude that, under “dual” tasking conditions, every subject performed three processes simultaneously: (i) a motor task (use of lower limbs, walking), (ii) a motor task (use of upper limbs, checking boxes) or an executive task (subtraction of serial 7 s), and (iii) an executive task (division of attention). According to this mechanistic model, either *two* motor tasks and *one* executive function task, or *one* motor task and *two* executive function tasks were simultaneously performed. Dual task costs of poor TMT performers were not different from good TMT performers when performing two motor and one executive function tasks simultaneously. This may be due to simplicity of the tasks; however this does not explain why none of the tasks was prioritized. We hypothesize that, in this particular situation, persons with poor executive function have sufficient capacity to divide attention appropriately. Contrary, dual task costs were higher in poor performers when performing *one* motor and *two* executive function tasks which affected the lower limb motor task, and the dividing attention task (but not the serial 7 s subtraction task). Thus, subjects with poor executive function capabilities may suffer from a bottleneck when performing two executive functions simultaneously. In this situation, these subjects prioritize the subtracting serial 7 s task (but obviously not the dividing attention task) at the expense of the motor task. From a clinical point of view this may be of relevance: Older persons with poor cognitive flexibility and working memory may be at particular risk for walking problems and falls under dual tasking situations which include an executive task not only because they are more prone to bottleneck situations per se, but also because of deteriorated prioritization capabilities. Our hypothesis is corroborated by two recent studies: Parkinson disease patients [10] and elderly fallers [11] have been shown to perform a secondary task most accurately at the expense of walking velocity. In addition, slowing of walking speed during secondary tasks can increase balance demands due to an increase of time spent for balancing the body over the stance leg [20], [21].

Interestingly, box checking with crosses did not add relevant information. As recently discussed by Al-Yahya and colleagues [22], this may not (only) be explained by the strong motor aspect of the task, but (also) by the observation that cognitive tasks that involve external interfering factors (e.g. reaction time) seem to disturb gait performance less than those involving internal interfering factors (e.g. mental tracking). In addition, the subtraction task may be considered more difficult than the box checking task and thus more informative regarding our working hypothesis. It has recently been shown that increased cognitive task complexity resulted in greater slowing of gait during dual tasking situations [23].

### Limitations

First, falls frequency of the study participants was not evaluated. Although there is convincing evidence that executive dysfunction is associated with occurrence of falls [12], [24] it would be interesting to compare this outcome parameter with prioritization aspects. Second, all groups performed better (faster) when subtracting serial 7 s under dual tasking, than under single tasking conditions. This may be best explained by learning effects (the dual task assessment was always performed after the single task assessment) or by a “rhythmicity” effect due to the simultaneously performed walking task. Nevertheless, this does not challenge the primary outcome of the study, i.e. the altered prioritization effect. Third, the cognitive test used for the assessment of cognitive flexibility gives rather crude information, and no test battery has been performed that more precisely differentiates between different forms of executive (dys)function. Future studies may thus use more detailed test batteries.

### Conclusion

This study demonstrates that poor cognitive flexibility and working memory in older subjects does not automatically lead to comparable dual task costs in the walking and non-walking task. Under most challenging dual tasking conditions, these subjects prioritize the cognitive task at the expense of the motor task. This “posture second” strategy may have effects on gait stability.
